# Specific Destruction of HIV Proviral p17 Gene in T Lymphoid Cells Achieved by the Genome Editing Technology

**DOI:** 10.3389/fmicb.2016.01001

**Published:** 2016-06-28

**Authors:** Tsunao Kishida, Akika Ejima, Osam Mazda

**Affiliations:** Department of Immunology, Kyoto Prefectural University of MedicineKamikyo, Japan

**Keywords:** HIV, genome edition, TALEN, T lymphoid cell line, provirus

## Abstract

Recent development in genome editing technologies has enabled site-directed deprivation of a nucleotide sequence in the chromosome in mammalian cells. Human immunodeficiency (HIV) infection causes integration of proviral DNA into the chromosome, which potentially leads to re-emergence of the virus, but conventional treatment cannot delete the proviral DNA sequence from the cells infected with HIV. In the present study, the transcription activator-like effector nucleases (TALENs) specific for the HIV p17 gene were constructed, and their activities to destroy the target sequence were evaluated. SSA assay showed a high activity of a pair of p17-specific TALENs. A human T lymphoid cell line, Jurkat, was infected with a lentivirus vector followed by transfection with the TALEN–HIV by electroporation. The target sequence was destructed in approximately 10–95% of the p17 polymerase chain reaction clones, and the efficiencies depended on the Jurkat–HIV clones. Because p17 plays essential roles for assembly and budding of HIV, and this gene has relatively low nucleotide sequence diversity, genome editing procedures targeting p17 may provide a therapeutic benefit for HIV infection.

## Introduction

Human immunodeficiency virus type 1 (HIV-1) causes latent infection in CD4^+^ T cells and macrophages, in which HIV-1 provirus DNA is integrated into the chromosomes. The viral genome is stably maintained in the cells and segregated into their progenies. Anti-HIV medicines such as protease inhibitors and reverse transcriptase inhibitors drastically improved prognosis of HIV-1-infected patients, by interfering with viral amplification (Pierson et al., 2000; Datta et al., 2016). However, perfect cure of HIV infection has not been achieved, because provirus DNA cannot be eliminated from the chromosomes of the infected cells by the present therapies.

Recent genome editing technologies including the zinc finger nuclease, transcription activator-like effector nucleases (TALEN), and clustered regularly interspaced short palindromic repeat (CRISPR)/Cas9 have made it possible to efficiently induce specific alteration or truncation of target nucleotide sequence in the genomic DNA of mammalian cells (Jinwei et al., 2015; Maeder and Gersbach, 2016; Mei et al., 2016). Such technologies may realize novel therapeutic procedures against various genetic diseases by replacing and modifying the genes responsible for the pathogenesis (Jang et al., 2016). Among them, TALEN is an artificial fusion enzyme composed of the nuclease domain and the DNA binding domain derived from the TALEs of the *Xanthomonas*. Any nucleotide sequence can be targeted by selecting the modules of the DNA-binding domains.

If provirus DNA is destroyed using these technologies, such procedures may provide a novel anti-HIV therapy that may not only eliminate the potential risk of HIV reactivation and AIDS onset but also relieve patients of potential adverse events and economic burden due to lifelong medication. The transactivation response element (TAR) sequence may be a suitable target (Ebina et al., 2015; Strong et al., 2015), while another appropriate target may be a coding sequence for an essential viral structural protein, because truncation of only a small number of nucleotides in the coding sequence may result in deletion of critical amino acid residue(s) or frameshift mutation.

In this context, we constructed TALENs that target the HIV Gag p17 gene, and examined if the p17 coding sequence in the chromosome of HIV-infected cells can be destroyed by transducing the recombinant TALEN vector.

## Materials and Methods

### TALEN Vectors

**FIGURE 1:**
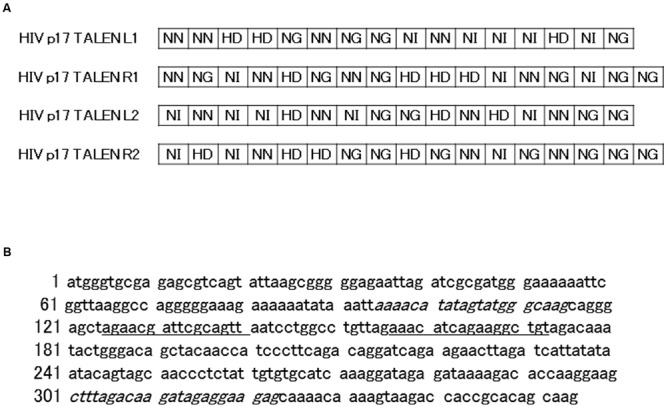
**Structure and target sequences of Human immunodeficiency (HIV) transcription activator-like effector nucleases (TALENs). (A)** The amino acid sequences of the repeat-variable di-residues (RVDs) of the indicated TALENs are shown with one-letter symbols. **(B)** The p17 gene sequence are shown. Underlined letters (125–140 and 157–173) represent target sites of HIV 1 and 2 TALENs, respectively, while italic letters represent forward and reverse polymerase chain reaction (PCR) primers (95–115, and 301–323, respectively) used for amplification of the p17 gene sequence from chromosomal DNA of Jurkat–HIV cells.

Two pairs of TALENs, i.e., HIV TALEN 1 (HIV TALEN 1 L and R) and HIV TALEN 2 (HIV TALEN 2 L and R) (**Figure 1A**) were designed to recognize p17 gene sequence (**Figure 1B**), based on the TAL Effector Nuclotide Targeter software.^1^ The TALEN vectors were constructed using the Golden Gate reaction as described previously.

### Single-Strand Annealing (SSA) Assay

**FIGURE 2:**
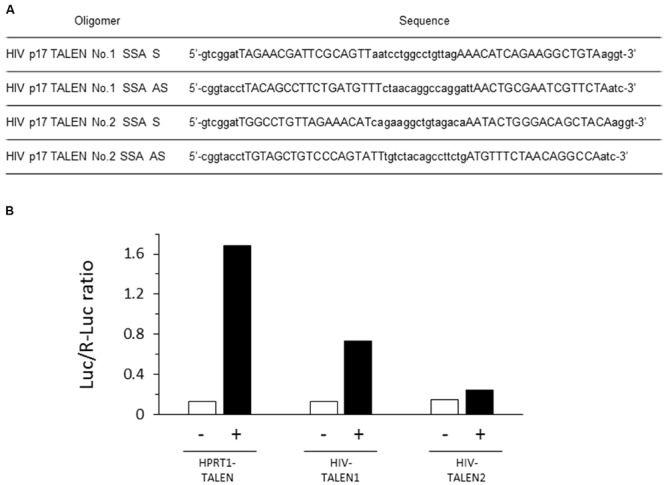
**Efficient destruction of p17 sequence by the HIV-1 TALEN. (A)** Sequences of the oligomers used in the SSA assay. **(B)** Each target sequence was inserted into pGL4-SSA reporter plasmid. These reporter plasmids (+) or empty pGL4-SSA (-) was co-transfected with the indicated TALEN expression vector and pRL-CMV reference plasmid into the 293TN cells. Twenty-four hours later, dual-Glo luciferase assay was performed to evaluate relative destruction of the target sequence. Luc/R-Luc ratios are shown. In the presence of HPRT1–TALEN, the HPRT target sequence was efficiently destroyed, resulting in high rate of R-Luc activity relative to Luc. HIV–TALEN1 destroyed its target sequence at an intermediate level, whereas HIV–TALEN2 showed very low destruction activity.

Single-strand annealing assay was performed as described with slight modification (Sakuma et al., 2013). Two pairs of oligonucleotides, HIV p17 TALEN No.1 SSA S and AS, and HIV p17 TALEN No. 2 SSA S and AS (**Figure 2A**), were annealed to form double strand oligonucleotides, which were subsequently inserted into pGL4-SSA reporter plasmid that had been digested by BsaI endonuclease. The resultant plasmids were named pGL4-SSA-HIV_1 and 2. 293TN cells were seeded into 96-well plates at a density of 6 × 10^4^/well. On the next day, cells were co-transfected with 20 ng of TALEN plasmid, 10 ng of pGL4-SSA, and 2 ng of pRL-CMV vector as an internal control by means of X-treme GENE 9 DNA Transfection Reagent (Roche). As a positive control, other aliquot of the cells were co-transfected with HPRT1 TALEN L1, HPRT1 TALEN R1, and pGL4-SSA-HPRT1 (Sakuma et al., 2013). Twenty-four hours later, dual luciferase assay was performed using Dual-Glo luciferase assay system (Promega) and GENios (TECAN Inc.) device.

### Lentivirus Vector

pGreen-puro vectors were purchaced from SBI. 293TN packaging cells (3 × 10^6^) were plated on geratin-coated 100 mm dishes and cultured overnight. They were co-transfected with pGreen-puro, pVSV-G, pPACKH1-REV, and pPACKH1-GAG using the X-treme Gene 9 transfection reagent (Roche Applied Science, Penzberg, Germany) diluted in Opti-MEM. Twenty-four hours later, the culture supernatant was replaced by antibiotic-free culture medium. After culturing for another 24 h, the supernatant was collected and filtered through a 0.45 μm pore-size filter.

### Cells, Infection, and Transfection

Jurkat, a human T cell leukemia cell line, was cultured in the RPMI1640 medium supplemented with 100 U/ml penicillin, 100 μg/ml streptomycin, and 10% FBS (Mazda et al., 1997). They were seeded onto culture dishes at a density of 1 × 10^6^ cells/mL. On the next day, cells were transduced with the lentiviral vector in the presence of 4 μg/mL polybrene (day 0). On day 1, cells were reseeded onto 96 well plates at 0.3 cells/well. After culturing with 1 μg/mL puromycin, three drug-resistant cell colonies were randomly chosen and named Jurkat–HIV P1–P3. The colonies were picked up and further cultured in puromycin-free medium. TALEN expression vectors were then transfected into the cells by electroporation (0.25 μg each of HIV TALEN 1 L and R vectors for 10^5^ cells).

### PCR and DNA Sequencing

Four days after the transfection, DNA was extracted from the cells. Polymerase chain reaction (PCR) was performed to amplify the p17 gene sequence using the primers shown in the **Figure 1B**. The resultant PCR fragments were inserted into a TA cloning vector, MD20, which were subsequently transformed into competent *Escherichia coli*. After seeding onto ampicillin-containing agar plates, twenty colonies were picked up. The sequence of each PCR clone was determined by standard procedure using the SP6 and M13 primers.

## Results

The HIV TALENs 1 and 2 were prepared to target HIV gag p17 gene (**Figure 1**) and the activities of the TALENs were assessed by the SSA assay. The results are shown in **Figure 2B**. The HIV TALENs 1 and 2 exhibited approximately 43 and 15% of activities compared with that of the positive control TALEN that were specific for hypoxanthine–guanine phosphoribosyl transferase (HPRT) sequence (Sakuma et al., 2013). Based on the results, we decided to use the HIV TALEN 1 that digests the HIV gag p17 gene sequence more efficiently than the HIV TALEN 2 in the following experiments.

The T lymphoma cell line, Jurkat, was infected with a lentivirus vector. After selection with puromycin, the resultant clones were expected to possess the lentivirus sequence integrated in their chromosomes. Thus, the lentivirus-infected Jurkat cells were regarded as a model of the T cells latently infected with HIV. Three clones (namely, Jurkat–HIV clone P1–P3) were transfected with the HIV TALEN 1, and nucleotide sequences of the target regions in PCR-amplified clones were determined.

The results are shown in **Tables 1–3**. In the Jurkat–HIV clone P1, 19 out of 20 PCR clones showed truncation of the p17 sequence (**Table 1**). The truncated sites spanned 7–22 nucleotides. Therefore, the HIV TALEN 1 cleaved the HIV sequence that had been integrated in the chromosome of Jurkat–HIV clone P1 at quite high efficiency. In contrast, nucleotide deletion was seen in only two out of twenty PCR clones derived from the Jurkat–HIV clone P2 (**Table 2**). Seven nucleotides were lacking in the p17 gene sequence in the two PCR clones. Meanwhile, a half of PCR clones (10 out of 20) derived from the Jurkat–HIV clone P3 possessed truncated p17 gene sequence (**Table 3**).

**Table 1:** Specific truncation of chromosomal DNA at p17 gene in Jurkat–HIV clone P1.

Control	agaacgattcgcagttaatcctggcctgttagaaacatcagaaggctgt
1	agaacgattcgca--------ctggcctgttagaaacatcagaaggctgt
2	agaacgattcgca--------ctggcctgttagaaacatcagaaggctgt
3	agaacgattcgca--------ctggcctgttagaaacatcagaaggctgt
4	agaacgattcgca--------ctggcctgttagaaacatcagaaggctgt
5	agaacgattcgca--------ctggcctgttagaaacatcagaaggctgt
6	agaacgattcgca--------ctggcctgttagaaacatcagaaggctgt
7	agaacgattcgca--------ctggcctgttagaaacatcagaaggctgt
8	agaacgattcgca--------ctggcctgttagaaacatcagaaggctgt
9	agaacgattcgca--------ctggcctgttagaaacatcagaaggctgt
10	agaacgattcgca--------ctggcctgttagaaacatcagaaggctgt
11	agaacgatt---------------------------gaaacatcagaaggctgt
12	agaacgatt---------------------------gaaacatcagaaggctgt
13	agaacgatt---------------------------gaaacatcagaaggctgt
14	agaacgatt---------------------------gaaacatcagaaggctgt
15	agaacgatt---------------------------gaaacatcagaaggctgt
16	agaacgatt----- ----------------------gaaacatcagaaggctgt
17	agaacgattcgcagtt-----------------agaaacatcagaaggctgt
18	agaacgattcgcagtt-----------------agaaacatcagaaggctgt
19	agaacgattcgcagtt-----------------agaaacatcagaaggctgt
20	agaacgattcgcagttaatcctggcctgttagaaacatcagaaggctgt

*Nucleotide sequences of 20 clones (No. 1–20) derived from PCR-amplified fragments of p17 gene region in the chromosomal DNA of Jurkat–HIV clone P1 are shown. Control at the top represents wild type p17 sequence.*

**Table 2:** Specific truncation of chromosomal DNA at p17 gene in Jurkat–HIV clone P2.

Control	agaacgattcgcagttaatcctggcctgttagaaacatcagaaggctgt
1	agaacgattcgca--------ctggcctgttagaaacatcagaaggctgt
2	agaacgattcgca--------ctggcctgttagaaacatcagaaggctgt
3	agaacgattcgcagttaatcctggcctgttagaaacatcagaaggctgt
4	agaacgattcgcagttaatcctggcctgttagaaacatcagaaggctgt
5	agaacgattcgcagttaatcctggcctgttagaaacatcagaaggctgt
6	agaacgattcgcagttaatcctggcctgttagaaacatcagaaggctgt
7	agaacgattcgcagttaatcctggcctgttagaaacatcagaaggctgt
8	agaacgattcgcagttaatcctggcctgttagaaacatcagaaggctgt
9	agaacgattcgcagttaatcctggcctgttagaaacatcagaaggctgt
10	agaacgattcgcagttaatcctggcctgttagaaacatcagaaggctgt
11	agaacgattcgcagttaatcctggcctgttagaaacatcagaaggctgt
12	agaacgattcgcagttaatcctggcctgttagaaacatcagaaggctgt
13	agaacgattcgcagttaatcctggcctgttagaaacatcagaaggctgt
14	agaacgattcgcagttaatcctggcctgttagaaacatcagaaggctgt
15	agaacgattcgcagttaatcctggcctgttagaaacatcagaaggctgt
16	agaacgattcgcagttaatcctggcctgttagaaacatcagaaggctgt
17	agaacgattcgcagttaatcctggcctgttagaaacatcagaaggctgt
18	agaacgattcgcagttaatcctggcctgttagaaacatcagaaggctgt
19	agaacgattcgcagttaatcctggcctgttagaaacatcagaaggctgt
20	agaacgattcgcagttaatcctggcctgttagaaacatcagaaggctgt

*Nucleotide sequences of 20 clones (No. 1–20) derived from PCR-amplified fragments of p17 gene region in the chromosomal DNA of Jurkat–HIV clone P2 are shown. Control at the top represents wild type p17 sequence.*

**Table 3:** Specific truncation of chromosomal DNA at p17 gene in Jurkat–HIV clone P3.

Control	agaacgattcgcagttaatcctggcctgttagaaacatcagaaggctgt
1	agaacgattcgca--------ctggcctgttagaaacatcagaaggctgt
2	agaacgattcgca--------ctggcctgttagaaacatcagaaggctgt
3	agaacgattcgca--------ctggcctgttagaaacatcagaaggctgt
4	agaacgattcgca--------ctggcctgttagaaacatcagaaggctgt
5	agaacgatt---------------------------gaaacatcagaaggctgt
6	agaacgatt---------------------------gaaacatcagaaggctgt
7	agaacgatt---------------------------gaaacatcagaaggctgt
8	agaacgatt---------------------------gaaacatcagaaggctgt
9	agaacgattcgcagtt-----------------agaaacatcagaaggctgt
10	agaacgattcgcagtt-----------------agaaacatcagaaggctgt
11	agaacgattcgcagttaatcctggcctgttagaaacatcagaaggctgt
12	agaacgattcgcagttaatcctggcctgttagaaacatcagaaggctgt
13	agaacgattcgcagttaatcctggcctgttagaaacatcagaaggctgt
14	agaacgattcgcagttaatcctggcctgttagaaacatcagaaggctgt
15	agaacgattcgcagttaatcctggcctgttagaaacatcagaaggctgt
16	agaacgattcgcagttaatcctggcctgttagaaacatcagaaggctgt
17	agaacgattcgcagttaatcctggcctgttagaaacatcagaaggctgt
18	agaacgattcgcagttaatcctggcctgttagaaacatcagaaggctgt
19	agaacgattcgcagttaatcctggcctgttagaaacatcagaaggctgt
20	agaacgattcgcagttaatcctggcctgttagaaacatcagaaggctgt

*Nucleotide sequences of 20 clones (No. 1–20) derived from PCR-amplified fragments of p17 gene region in the chromosomal DNA of Jurkat–HIV clone P3 are shown. Control at the top represents wild type p17 sequence.*

## Discussion

In the present study, the HIV TALEN 1 that we constructed may highly efficiently edit the p17 sequence as suggested by the SSA assay, although the reason why the other TALEN was less efficient remains unknown. The TALEN induced cleavage and truncation of the target sequence in the Jurkat–HIV cells, leading to destruction of the coding sequence of the p17 gene. In HIV-infected cells, the HIV-1 matrix protein p17 is excised by proteolysis from the N-terminus of the Gag polyprotein, and bring the Gag to the host cell membrane via its N-terminal myristoyl group (Freed, 1998). Because the p17 is essential for the assembly and budding of HIV virion, deletion of this gene from the chromosome may bring remarkable therapeutic benefits (Ellenrieder et al., 2004). The p17 gene has less sequence variation among virus subtypes compared with the env genes (Brown and Monaghan, 1988; Markham et al., 1995). Thus, the p17 gene may be suitable as the target of the gene editing therapeutic strategy.

We tested three Jurkat–HIV clones that we established by limiting dilution after the infection with the lentivirus vector. Interestingly, the three clones underwent p17 truncation at different rates ranging from 10 to 95%. The variation among the clones may be due to different integration sites of the provirus sequence in the chromosomes, and/or difference in the copy number of the integrated provirus per cell. Alternatively, efficiency of electro-transfection of the TALEN vector may differ among the Jurkat–HIV cell clones, resulting in different proportion of the cells that sufficiently expressed TALEN among the cell population. In the present study, the TALEN vector was transfected into the cells once by electroporation. If the electroporation is repetitively performed, the efficiency of transfection may be elevated, resulting in a higher rate of p17 gene truncation. Some delivery procedure other than electroporation may also be used to transfer the TALEN vector into the cells to increase the efficiency of the genome edition. A lentivirus vector may be quite suitable for the delivery of a TALEN vector into T cells in patients in whom HIV is latently infected.

## Author Contributions

TK: Conception and design, Provision of study materials, Data analysis, and interpretation; AE: Data analysis and interpretation, OM: Manuscript writing, Administrative support, and Final approval of manuscript.

## Conflict of Interest Statement

The authors declare that the research was conducted in the absence of any commercial or financial relationships that could be construed as a potential conflict of interest.

## Footnotes

1 https://tale-nt.cac.cornell.edu/
